# Exploring rare coding variants in UK biobank: preliminary associations with motor neuron disease

**DOI:** 10.3389/fnagi.2025.1735522

**Published:** 2026-01-09

**Authors:** Zhen Hu, Jing-jin Wan, Qin-qin Yan, Yu Fan, Jun Liu

**Affiliations:** 1 Department of Neurology and Institute of Neurology, Ruijin Hospital, Shanghai Jiao Tong University School of Medicine, Shanghai, China; 2 Department of Surgery, Renji Hospital, Shanghai Jiao Tong University School of Medicine, Shanghai, China; 3 Department of Radiology, Ruijin Hospital, Shanghai Jiao Tong University School of Medicine, Shanghai, China

**Keywords:** motor neuron disease, protein-truncating variants, rare variants, risk factors, UK biobank

## Abstract

**Introduction:**

Previous studies have illuminated a significant genetic component in motor neuron disease (MND) pathogenesis, with several causative genes identified. However, a substantial proportion of MND cases remain genetically unexplained, particularly regarding the comprehensive contribution of rare, high-impact variants across the exome.

**Methods:**

Leveraging whole-exome sequencing data from nearly half a million UK Biobank participants, we systematically investigated the association between high-confidence protein-truncating variants (HC PTVs) and MND risk in a Caucasian subset. Our large-scale gene-based association analysis utilized REGENIE software and LOFTEE-defined HC PTVs.

**Results:**

We identified significant preliminary associations between HC PTVs in 14 genes and an increased risk of MND. Notably, while NEK1 has been previously implicated in ALS, the remaining 13 genes (*BLVRB*, *KLHL32*, *RIMS2*, *DYDC2*, *DCBLD1*, *ANXA4*, *COMP*, *TRIM42*, *ANO4*, *NFX1*, *CFAP206*, *CKAP2L*, and *ANGPTL4*) show preliminary associations as novel candidate loci for the disease. Functional enrichment analyses further indicated that these genes are significantly involved in critical biological pathways, including collagen-containing extracellular matrix organization and ciliary function. Furthermore, tissue specificity analysis highlighted a strong enrichment of these genes’ expression in brain regions, with the hypothalamus showing the highest specificity.

**Discussion:**

These findings suggest a potential expansion of the known genetic landscape of MND, and highlight novel biological pathways implicated in its pathogenesis. This study underscores the power of large-scale population genetics in uncovering critical disease mechanisms and offers new avenues for mechanistic research and therapeutic development for MND, pending independent validation.

## Introduction

Motor neuron disease (MND) represents a group of devastating, progressive neurodegenerative disorders characterized by the selective degeneration of upper and/or lower motor neurons (Foster and Salajegheh, 2019; Malkki, 2016; Babazadeh et al., 2023). This relentless progression ultimately leads to muscle weakness, atrophy, paralysis, and typically respiratory failure and death (Winhammar et al., 2005; Rocha et al., 2005). The most common and rapidly progressive form is amyotrophic lateral sclerosis (ALS), but the clinical spectrum also encompasses progressive muscular atrophy (PMA) and primary lateral sclerosis (PLS), underscoring the disease’s significant clinical and pathological heterogeneity (Feldman et al., 2022; Angilletta et al., 2023; Turner et al., 2020). Globally, MND imposes an immense burden on patients, their families, and healthcare systems, with a challenging prognosis and a critical lack of effective disease-modifying treatments currently available (Rizea et al., 2024; Niedermeyer et al., 2019; Owens, 2017).

Genetics plays an increasingly recognized and pivotal role in MND pathogenesis. While approximately 5 to 7% of MND cases are familial, a substantial genetic contribution is also evident in sporadic forms, highlighting a complex interplay of genetic predisposition and environmental factors (Foster and Salajegheh, 2019; de Boer et al., 2024; Benatar et al., 2009). The identification of key causative genes such as *C9orf72* (Mizielinska et al., 2025), *SOD1* (Berdyński et al., 2022), *TARDBP* (Balendra et al., 2025), and *FUS* (Mackenzie et al., 2010) has advanced our understanding of MND’s intricate pathological mechanisms, implicating diverse pathways including RNA processing, protein aggregation, and nucleocytoplasmic transport (Beers and Appel, 2019; Tolochko et al., 2025). Despite these significant advancements, a large proportion of MND cases still lack a defined genetic etiology (Malkki, 2016; Chaudhary et al., 2022). Previous genetic studies, while foundational, may have faced limitations due to insufficient sample sizes, inadequate power to detect rare variants, or less comprehensive analytical methodologies, leaving many genetic underpinnings of MND yet to be uncovered.

Whole exome sequencing (WES) has emerged as a powerful and cost-effective tool in genetic research, offering distinct advantages over traditional genome-wide association studies (GWAS) for detecting rare coding region variants. WES’s ability to identify high-impact, low-frequency variants makes it particularly suited for exploring novel disease-associated genes and genetic modifiers that might be missed by common variant approaches. This technology allows for a targeted yet comprehensive examination of the protein-coding regions of the genome, where a significant proportion of Mendelian disease-causing mutations reside.

The UK Biobank is an unparalleled resource for genetic association studies, comprising a prospective cohort of 500,000 participants with extensive phenotypic data, including detailed health records, and high-quality WES data (Allen et al., 2024; Backman et al., 2021; Van Hout et al., 2020). This large-scale dataset significantly boosts the statistical power to uncover novel genetic associations and minimize false positives by enabling robust statistical analyses that account for confounding factors. Crucially, the inclusion of over 800 individuals diagnosed with MND within this well-characterized cohort provides a unique and invaluable opportunity to delve deeper into the genetic landscape of the disease.

Building upon these strengths, our study aimed to leverage the UK Biobank’s WES data to explore potential new genes associated with an increased risk of MND (Backman et al., 2021; Van Hout et al., 2020). Utilizing rigorous gene-based association testing with REGENIE (Mbatchou et al., 2021) software and focusing on high-confidence protein-truncating variants (HC PTVs) as defined by LOFTEE (Karczewski et al., 2020), we systematically screened for genetic predispositions within the Caucasian subset of this cohort. Here, we report the preliminary identification of 18 genes whose HC PTVs show significant association with an elevated risk of MND. Notably, while *NEK1* is a previously implicated locus, the remaining 17 genes represent new candidate associations that warrant independent validation. Functional and tissue specificity analyses of these candidate genes suggest the involvement of biological pathways, including the collagen-containing extracellular matrix and cilium, and highlight strong expression patterns in brain regions, particularly the hypothalamus. These preliminary findings contribute to the genetic understanding of MND and provide new avenues for mechanistic research.

## Methods

### Ethics

The UK Biobank is a large-scale, prospective cohort study that comprehensively collects phenotypic and genetic data from approximately 500,000 participants, who were aged 38 to 72 years at the time of their enrollment (Bycroft et al., 2018). Ethical approval for the study was granted by the North West Multi-Centre Research Ethics Committee,^1^ and all participants provided written informed consent. This overarching ethical clearance allows researchers to utilize UK Biobank data without seeking separate approval for individual studies. Our specific investigation was conducted under UK Biobank application number 162635.

### UK biobank data processing and quality control

We defined our MND cohort using algorithmically-derived outcomes provided directly by UK Biobank (Data-Field 42,028 and Data-Field 42,029). The dataset included a range of diagnostic categories: 51 self-reported cases, 372 cases with a primary hospital diagnosis, 119 cases with a primary diagnosis on death certificates, 273 cases with a secondary hospital diagnosis, and 10 cases where MND was a contributory cause of death.

For our analysis, we leveraged whole-exome sequencing (WES) data from 469,589 individuals from the UK Biobank (Backman et al., 2021). We initially excluded participants based on standard quality control (QC) criteria, including those exhibiting excess heterozygosity, individuals with ≥5% autosomal variant missingness on genotyping arrays, or those not categorized as part of the phased samples subset (Bycroft et al., 2018). WES data, aligned to GRCh38, were provided as population- or individual-level files and accessed via the UK Biobank Research Analysis Platform (RAP). In addition to the existing QC measures applied by the data providers (Backman et al., 2021), we implemented several supplementary QC procedures. We used ‘bcftools v1.14 norm’ (Danecek et al., 2021) to split multi-allelic sites and to perform left-correction and normalization of indels. We then filtered out variants that did not meet our stringent criteria: (1) a read depth of less than 7; (2) a genotype quality score below 20; and (3) a binomial test *p*-value for alternative allele reads versus reference allele reads of ≤0.001 for heterozygous genotypes. For indel genotypes, we applied a more stringent criterion, retaining only variants with a read depth of at least 10 and a genotype quality of at least 20. Variants that failed to meet these thresholds were designated as missing. Subsequently, any variant with more than 50% missing genotypes was excluded from all downstream analyses (Gardner et al., 2022).

The remaining variants were annotated using Ensembl Variant Effect Predictor (VEP v104) (McLaren et al., 2016), with the ‘-everything’ flag enabled and additional plugins for REVEL (Ioannidis et al., 2016), CADD (Rentzsch et al., 2019), and LOFTEE (Karczewski et al., 2020). AlphaMissense scores were integrated by downloading data from the corresponding GitHub repository (Cheng et al., 2023). A single Ensembl transcript was prioritized for each variant based on a hierarchical scheme: protein-coding transcripts were preferred, followed by MANE select v0.97 transcripts (Morales et al., 2022), and finally, the VEP canonical transcript. Variant consequences were ranked by severity as defined by VEP. Stop-gained, splice-site disrupting, and frameshift variants were aggregated into a single protein-truncating variant (PTV) category after being filtered by LOFTEE to minimize false positives (Karczewski et al., 2020). Annotations for missense and synonymous variants were adopted directly from VEP. Our primary analysis cohort consisted of individuals of ‘white European’ ancestry, excluding those who self-identified as belonging to other ancestries via a questionnaire, as well as participants who had withdrawn consent from the study. This stringent selection process resulted in a final cohort of 393,746 individuals.

To prepare for sensitivity analyses, we identified individuals with known MND- or ALS-associated pathogenic variants. We obtained a list of 627 reported pathogenic/likely pathogenic MND/ALS variants from the ClinVar database^2^ and found that these were carried by 1,634 individuals in our dataset, eight of whom were MND cases. We also calculated the repeat size of the *C9ORF72* GGGGCC expansion using ExpansionHunter (Dolzhenko et al., 2019) on individual-level whole-genome sequencing (WGS) data. The vast majority (>95%) of neurologically healthy individuals have ≤11 hexanucleotide repeats in the *C9ORF72* gene (Rutherford et al., 2012). As the pathological repeat-length threshold has not been definitively established, we used an arbitrary cutoff of 30 repeats, which is common practice in most studies (Balendra and Isaacs, 2018). This led to the exclusion of 849 individuals exceeding this threshold, 67 of whom were MND cases. Six individuals were found to harbor both ClinVar-reported pathogenic/likely pathogenic mutations and over 30 *C9ORF72* GGGGCC repeats. To account for potential relatedness, we identified all possible first-degree relatives using a correlation coefficient exceeding 0.49 from the Genetic Relationship Matrix (GRM) for all samples with WES data.

### Gene-burden testing in UK biobank

REGENIE v4.1 (Mbatchou et al., 2021) served as our primary analytical tool for conducting the gene-burden test. To initiate our analysis, we first constructed a null model by querying a set of genotypes with a minor allele count (MAC) greater than 100, derived from genotyping arrays for individuals with WES data. To enable gene-level testing, we collapsed variants into unified ‘mask’ genotypes for association analysis, aligning with REGENIE’s documentation.

We defined high-confidence (HC) PTVs as stop-gained, splice-site disrupting, and frameshift variants that had been filtered by LOFTEE to minimize false positives. We then generated masks for these HC PTVs with a minor allele frequency (MAF) less than 0.1%, as determined by LOFTEE. Additionally, masks were created for missense variants using various established pathogenicity prediction thresholds: CADD (>20), REVEL (>0.7 and >0.5), and AlphaMissense (>0.9, >0.7, and >0.56). We subsequently used REGENIE to analyze phenotypes using its default parameters. Our models included age, sex, and the first 10 principal components (PCs) as calculated by Bycroft et al. (2018) as covariates. We also performed SKAT, SKAT-O, and SKAT-ACAT analyses using REGENIE (Zhao et al., 2024; Wu et al., 2011; Lee et al., 2012), applying the identical variant filtering and covariate inclusion as previously described. For all gene-burden tests, a Bonferroni-adjusted *p*-value threshold was set at 2.5 × 10^−6^ (derived from 0.05/20,000). Odds ratios (ORs) were calculated using logistic regression.

## Results

We included a total of 663 MND cases (age 60.83 ± 6.57 years, 44.19% Female) and 393,083 controls (age 56.91 ± 8.00 years, 54.04% Female) of European ancestry with available WES data in our primary analysis. Our gene-based association analysis revealed that protein-truncating variants (PTVs) in 18 genes (*BLVRB*, *KLHL32*, *NEK1*, *RIMS2*, *DYDC2*, *DCBLD1*, *ANXA4*, *SLC44A3*, *ATP10A*, *FRAS1*, *COMP*, *TRIM42*, *ANO4*, *NFX1*, *CFAP206*, *NLRP2*, *CKAP2L* and *ANGPTL4*) were significantly enriched in MND cases compared to controls (*P_SKAT-O_* < 2.5 × 10^−6^, Table 1).

**Table 1:** Gene-based burden analysis of high-confidence protein-truncating variants in candidate motor neuron disease risk genes.

Mask	Carrier (%)	Case (%)	Logistic regression		SKAT		
			OR(95%CI)	*p*	*P*_SKAT_	*P*_SKAT-O_	*P*_SKATO-ACAT_
Primary analysis	*N* = 393,083	*N* = 663					
*BLVRB*	111 (0.03%)	3 (0.45%)	18.64 (7.72–45.02)	7.85E-11	1.38E-15	**1.41E-15**	2.57E-15
*KLHL32*	67 (0.02%)	3 (0.45%)	30.80 (13.33–71.15)	1.04E-15	1.00E-14	**2.29E-15**	1.35E-15
*NEK1*	613 (0.16%)	9 (1.36%)	9.23 (5.29–16.10)	4.77E-15	6.36E-11	**6.75E-15**	1.39E-14
*RIMS2*	150 (0.04%)	3 (0.45%)	13.81 (5.39–35.41)	4.60E-08	1.80E-14	**3.17E-14**	4.06E-14
*DYDC2*	100 (0.03%)	3 (0.45%)	20.68 (8.02–53.37)	3.76E-10	6.39E-08	**4.18E-10**	2.28E-09
*DCBLD1*	242 (0.06%)	4 (0.6%)	11.03 (5.12–23.77)	9.05E-10	3.36E-08	**7.24E-10**	5.98E-10
*ANXA4*	168 (0.04%)	3 (0.45%)	12.34 (4.63–32.85)	4.96E-07	1.52E-09	**4.10E-09**	1.55E-09
*SLC44A3*	342 (0.09%)	4 (0.6%)	7.81 (3.01–20.26)	2.41E-05	5.66E-09	**1.68E-08**	9.89E-09
*ATP10A*	179 (0.05%)	3 (0.45%)	11.58 (4.53–29.58)	3.09E-07	4.16E-08	**2.14E-08**	2.66E-08
*FRAS1*	643 (0.16%)	7 (1.06%)	6.94 (3.52–13.69)	2.3E-08	3.38E-05	**2.44E-08**	1.20E-07
*COMP*	282 (0.07%)	3 (0.45%)	7.36 (2.66–20.38)	1.24E-04	6.99E-09	**2.56E-08**	1.41E-08
*TRIM42*	655 (0.17%)	4 (0.6%)	4.08 (1.65–10.05)	2.26E-03	1.77E-08	**4.62E-08**	4.40E-08
*ANO4*	167 (0.04%)	3 (0.45%)	12.41 (4.89–31.52)	1.19E-07	2.91E-07	**5.10E-08**	1.06E-07
*NFX1*	119 (0.03%)	3 (0.45%)	17.40 (5.61–53.96)	7.62E-07	7.62E-07	**7.62E-07**	7.62E-07
*CFAP206*	611 (0.16%)	4 (0.6%)	4.37 (1.57–12.21)	4.87E-03	2.95E-07	**8.29E-07**	6.73E-07
*NLRP2*	972 (0.25%)	7 (1.06%)	4.58 (2.29–9.19)	1.79E-05	1.67E-06	**1.28E-06**	1.11E-06
*CKAP2L*	258 (0.07%)	3 (0.45%)	8.04 (2.90–22.30)	6.20E-05	1.43E-06	**1.92E-06**	1.67E-06
*ANGPTL4*	382 (0.1%)	3 (0.45%)	5.43 (1.68–17.58)	4.73E-03	7.70E-07	**2.46E-06**	1.79E-06
Sensitivity analysis	*N* = 390,681	*N* = 588					
*BLVRB*	111 (0.03%)	3 (0.51%)	20.90 (8.85–49.33)	4.00E-12	1.47E-17	**1.49E-17**	2.77E-17
*KLHL32*	65 (0.02%)	3 (0.51%)	35.58 (15.82–79.99)	5.63E-18	1.05E-16	**1.40E-17**	8.26E-18
*NEK1*	314 (0.08%)	4 (0.68%)	9.53 (4.05–22.42)	2.38E-07	5.36E-07	**4.90E-08**	1.01E-07
*RIMS2*	150 (0.04%)	3 (0.51%)	15.48 (6.17–38.82)	5.19E-09	1.59E-16	**3.41E-16**	3.76E-16
*DYDC2*	99 (0.03%)	3 (0.51%)	23.42 (9.31–58.92)	2.09E-11	8.54E-09	**2.31E-11**	1.39E-10
*DCBLD1*	242 (0.06%)	4 (0.68%)	12.36 (5.85–26.12)	4.43E-11	2.64E-09	**3.39E-11**	2.37E-11
*ANXA4*	168 (0.04%)	3 (0.51%)	13.83 (5.37–35.60)	5.20E-08	4.62E-11	**1.27E-10**	4.80E-11
*SLC44A3*	337 (0.09%)	2 (0.34%)	4.93 (0.97–25.04)	5.46E-02	2.80E-04	5.41E-04	5.51E-04
*ATP10A*	179 (0.05%)	2 (0.34%)	9.26 (3.21–26.71)	3.78E-05	3.70E-07	3.82E-07	5.68E-07
*FRAS1*	638 (0.16%)	5 (0.85%)	5.74 (2.45–13.45)	5.78E-05	1.24E-03	6.59E-05	1.60E-04
*COMP*	282 (0.07%)	3 (0.51%)	8.25 (3.06–22.20)	2.98E-05	2.14E-10	**1.11E-09**	4.56E-10
*TRIM42*	651 (0.17%)	4 (0.68%)	4.60 (1.87–11.28)	8.61E-04	1.52E-09	**4.35E-09**	3.89E-09
*ANO4*	166 (0.04%)	3 (0.51%)	13.99 (5.58–35.08)	1.83E-08	6.06E-08	**7.56E-09**	1.78E-08
*NFX1*	118 (0.03%)	3 (0.51%)	19.66 (6.51–59.38)	1.28E-07	1.28E-07	**1.28E-07**	1.28E-07
*CFAP206*	606 (0.16%)	4 (0.68%)	4.94 (1.81–13.48)	1.82E-03	3.29E-08	**1.06E-07**	7.50E-08
*NLRP2*	963 (0.25%)	5 (0.85%)	3.80 (1.63–8.87)	2.03E-03	2.95E-04	4.09E-04	3.00E-04
*CKAP2L*	257 (0.07%)	3 (0.51%)	9.04 (3.32–24.63)	1.63E-05	2.38E-07	**3.07E-07**	2.71E-07
*ANGPTL4*	382 (0.1%)	3 (0.51%)	6.09 (1.94–19.09)	1.95E-03	7.65E-08	**3.04E-07**	1.83E-07
Six more genes identified by sensitivity analysis							
*EPHX1*	235 (0.06%)	3 (0.51%)	9.89 (3.91–25.04)	1.31E-06	2.79E-06	**9.49E-07**	7.24E-07
*SELENOV*	228 (0.06%)	3 (0.51%)	10.20 (4.12–25.23)	5.11E-07	2.88E-05	**1.10E-06**	1.73E-06
*CDT1*	226 (0.06%)	3 (0.51%)	10.29 (3.19–33.18)	9.59E-05	1.02E-06	**1.17E-06**	1.22E-06
*ACTN3*	595 (0.15%)	4 (0.68%)	5.03 (1.99–12.74)	6.49E-04	9.91E-07	**2.35E-06**	1.61E-06
*USP16*	230 (0.06%)	3 (0.51%)	10.11 (3.89–26.25)	2.03E-06	1.71E-05	**2.42E-06**	5.05E-06
*MAJIN*	309 (0.08%)	4 (0.68%)	9.69 (3.78–24.79)	2.19E-06	2.46E-05	**2.46E-06**	7.28E-06

Bold values indicate statistical significance with *P_SKAT-O_* < 2.5 × 10^−6^ (Bonferroni-adjusted threshold).

Among these, PTVs in *BLVRB* exhibited the most significant association with MND, showing the strongest statistical evidence (OR = 18.64, 95% CI = 7.72–45.02, *P_GLM_* = 7.85 × 10^−11^, *P_SKAT-O_* = 1.41 × 10^−15^). As a well-established ALS gene (Mann et al., 2023; Noh et al., 2025; Rifai et al., 2025), PTVs in *NEK1* showed the highest carrier frequency in MND cases, detected in 9 out of 663 MND patients (1.36%) compared to 613 out of 393,083 controls (0.16%, OR = 9.23, 95% CI = 5.29–16.10, *P_GLM_* = 4.77 × 10^−15^, *P_SKAT-O_* = 6.75 × 10^−15^). *FRAS1* and *NLRP2* PTVs ranked second in carrier frequency among MND cases, with *FRAS1* PTVs observed in 7 out of 663 patients (1.06%) compared to 643 out of 393,083 controls (0.16%, OR = 6.94, 95% CI = 3.52–13.69, *P_GLM_* = 2.30 × 10^−8^, *P_SKAT-O_* = 2.44 × 10^−8^). *NLRP2* PTVs were found in 7 MND cases and 972 control individuals (0.25%, OR = 4.58, 95% CI = 2.29–9.19, *P_GLM_* = 1.79 × 10^−5^, *P_SKAT-O_* = 1.28 × 10^−6^). PTVs in *DCBLD1*, *SLC44A3*, *TRIM42*, and *CFAP206* were identified in 4 out of 663 MND cases (0.60%). PTVs in the remaining 10 genes (*KLHL32*, *RIMS2*, *DYDC2*, *ANXA4*, *ATP10A*, *COMP*, *ANO4*, *NFX1*, *CKAP2L*, and *ANGPTL4*) were identified in 3 out of 663 MND cases (0.45%) each, with ORs for PTVs in these genes ranging from 4.08 to 30.80. Genes with fewer than three PTV carriers were excluded from the analysis to ensure statistical robustness.

In contrast to PTVs, no significant association was observed between missense variants of the other 17 candidate genes and MND, irrespective of pathogenicity prediction thresholds (AlphaMissense > 0.9, > 0.7 or > 0.6; REVEL > 0.7 or > 0.5; CADD > 20) or sensitivity analyses (Supplementary Table S1). This suggests that the primary pathogenicity in these 17 candidate genes is likely attributed to PTVs rather than missense variants. However, for *NEK1*, missense variants of different masks (AlphaMissense > 0.9, > 0.7 or > 0.6; REVEL > 0.7 or > 0.5) were significantly enriched in MND cases compared to control individuals (*P_SKAT-O_* < 2.5 × 10^−6^, Supplementary Table S1), highlighting the high pathogenicity of *NEK1* missense variants and their significant contribution to MND pathogenesis.

To further strengthen the robustness of our findings, we performed sensitivity analyses by excluding individuals with known confounding genetic factors. First, 1,634 individuals harboring previously reported pathogenic or likely pathogenic mutations for MND or amyotrophic lateral sclerosis (ALS), according to the ClinVar database, were excluded. Second, given that GGGGCC repeat expansion in the *C9ORF72* gene is a frequent cause of ALS (Balendra and Isaacs, 2018; DeJesus-Hernandez et al., 2011; Todd and Paulson, 2013), we calculated repeat sizes using ExpansionHunter (Dolzhenko et al., 2019) for all individuals with available WGS data. An arbitrary cutoff of 30 GGGGCC repeats, commonly used in most studies (Balendra and Isaacs, 2018), was applied, leading to the exclusion of 849 individuals exceeding this threshold. Six individuals were found to harbor both ClinVar-reported pathogenic/likely pathogenic mutations and over 30 *C9ORF72* GGGGCC repeats.

After excluding a total of 2,652 individuals (those with reported ALS pathogenic/likely pathogenic mutations and/or *C9ORF72* GGGGCC repeat expansion), our sensitivity analysis was performed on a cohort of 588 MND cases (age 61.16 ± 6.50 years, 43.88% Female) and 390,681 controls (age 56.91 ± 8.00 years, 54.04% Female). This analysis revealed that PTVs in 14 out of the 18 genes identified in the primary analysis remained significantly enriched in MND cases compared to controls (*P_SKAT-O_* < 2.5 × 10^−6^, Table 1), except for *SLC44A3*, *ATP10A*, *FRAS1* and *NLRP2*. Furthermore, the sensitivity analysis identified six additional genes (*EPHX1*, *SELENOV*, *CDT1*, *ACTN3*, *USP16*, and *MAJIN*) whose PTVs were significantly enriched in MND cases (*P_SKAT-O_* < 2.5 × 10^−6^, Table 1), but not in controls. PTVs for these six genes were identified in three to four MND cases each. PTVs in *NEK1* remained significantly enriched in MND cases in the sensitivity analysis, with 4 out of 588 MND patients (0.68%) carrying PTVs compared to 314 out of 390,681 controls (0.08%, OR = 9.53, 95% CI = 4.05–22.42, *P_GLM_* = 2.38 × 10^−7^, *P_SKAT-O_* = 4.90 × 10^−8^). Consistent with the primary analysis, no significant association was observed between missense variants of the 20 candidate MND genes identified in the sensitivity analysis and MND, except for *NEK1* (Supplementary Table S1). For *NEK1*, missense variants of different masks (AlphaMissense > 0.9, > 0.7 or > 0.6; REVEL > 0.7 or > 0.5) continued to show significant enrichment in MND cases (*P_SKAT-O_* < 2.5 × 10^−6^, Supplementary Table S1).

**Figure 1:**
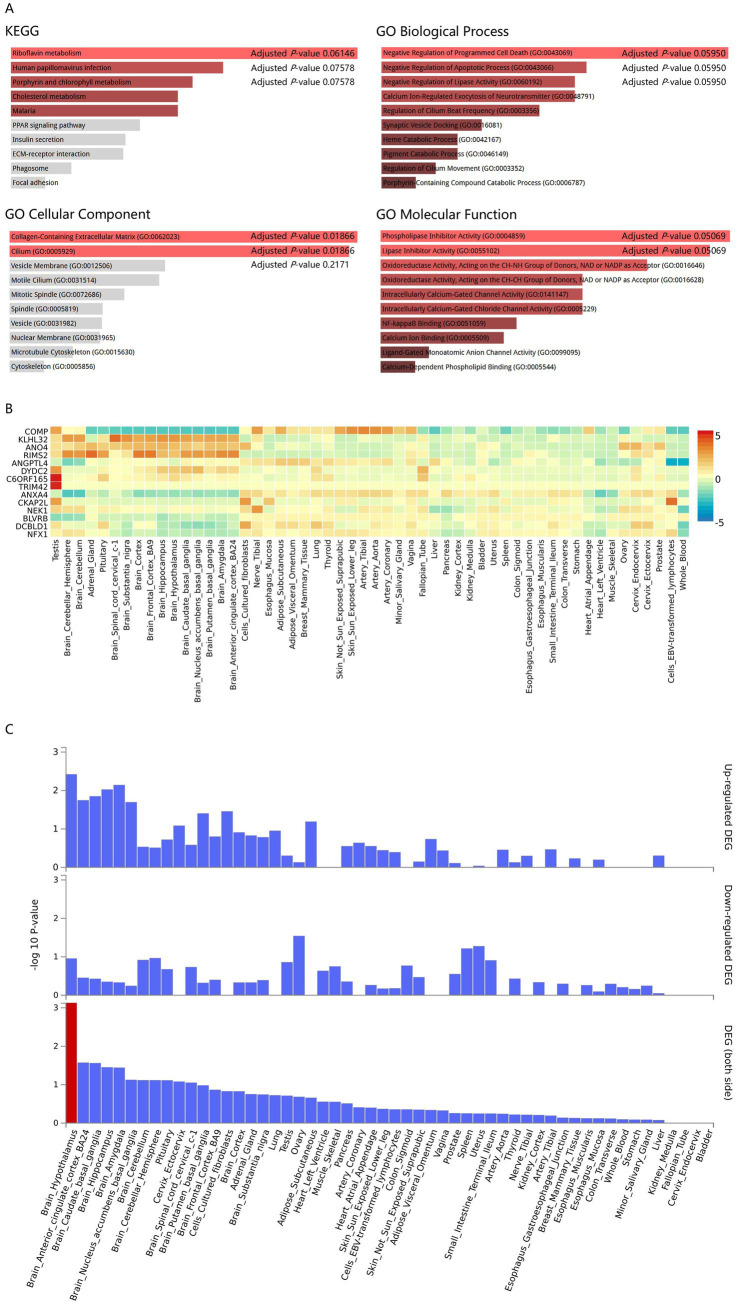
Functional enrichment and tissue-specific expression analyses of the 14 candidate MND genes identified through robust gene-burden testing. **(A)** Gene ontology (GO) and KEGG pathway enrichment analysis. The figure displays the significantly enriched GO cellular component (CC) terms (Bonferroni adjusted *p* < 0.05) for the 14 candidate MND genes (those remaining significant in both primary and sensitivity analyses: *BLVRB*, *KLHL32*, *RIMS2*, *DYDC2*, *DCBLD1*, *ANXA4*, *COMP*, *TRIM42*, *ANO4*, *NFX1*, *CFAP206*, *CKAP2L*, *NEK1*, and *ANGPTL4*). Significant enrichment was noted in the collagen-containing extracellular matrix (GO:0062023) and cilium (GO:0005929). These findings suggest the potential involvement of pathways related to structural integrity and primary ciliary signaling in MND pathogenesis. GO biological process (BP), molecular function (MF), and KEGG pathways did not show significant enrichment after correction. **(B)** Expression patterns across 54 tissues (GTEx v8). This heatmap illustrates the normalized expression level (TPM, transcripts per million) of the 14 candidate genes across 54 human tissue types profiled by the Genotype-Tissue Expression (GTEx) project. The color intensity reflects the relative expression level, with darker shades indicating higher expression. Genes are clustered based on their expression profiles, showing variable patterns. Notably, several genes (*KLHL32*, *ANO4*, *RIMS2*, *DYDC2*, and *CFAP206*) exhibit generally higher expression in various brain regions compared to non-brain tissues. **(C)** Tissue specificity analysis via differentially expressed genes (DEGs). This analysis evaluates the tissue specificity of the 14 candidate MND genes by assessing the enrichment of up-regulated differentially expressed genes (DEGs) in human tissues. The analysis revealed that up-regulated DEGs were predominantly enriched in brain regions. The total DEG analysis indicated that the brain hypothalamus exhibited the highest tissue specificity (Bonferroni adjusted *p*-value < 0.05, highlighted in red). This strong brain-specific expression pattern in the hypothalamus, a region critical for autonomic and metabolic regulation, suggests that genetic perturbations in these identified genes may have direct pathological consequences in the central nervous system.

Functional enrichment analysis (KEGG/GO) of the 14 candidate MND genes (from both primary and sensitivity analyses that remained significant, Figure 1A) showed no significant KEGG pathway enrichment (e.g., riboflavin metabolism adjusted *p* = 0.06146). Similarly, GO Biological Process (BP) and Molecular Function (MF) terms showed no significant enrichments. However, GO Cellular Component (CC) analysis revealed significant enrichments in collagen-containing extracellular matrix (GO:0062023, adjusted *p* = 0.01866) and cilium (GO:0005929, adjusted *p* = 0.01866). These findings suggest extracellular matrix remodeling and ciliary dysfunction as potential novel mechanisms in MND pathogenesis.

To further understand the expression patterns of these 14 MND candidate genes, we explored their tissue expression using the Genotype-Tissue Expression Project (GTEx, https://gtexportal.org/home/) database (GTEx Consortium, 2013) (Figure 1B). We found that expression levels of *COMP, ANXA4, CKAP2L, BLVRB, DCBLD1*, and *NFX1* were generally lower in brain regions compared to non-brain regions, while *KLHL32, ANO4, RIMS2, DYDC2*, and *CFAP206 (C6ORF165)* showed higher expression in brain regions. We then investigated the tissue specificity of these 14 candidate MND genes through differentially expressed gene (DEG) analysis in human tissues (Figure 1C). Our analysis revealed that up-regulated DEGs were predominantly enriched in brain regions. Furthermore, the total DEG analysis indicated that the brain hypothalamus, a region known for its crucial roles in energy metabolism, autonomic function, and neuroinflammation, exhibited the highest tissue specificity (significantly enriched DEG sets, Bonferroni adjusted *p*-value < 0.05, highlighted in red in Figure 1C). This strong brain-specific expression pattern, particularly in a functionally crucial region like the hypothalamus, points towards a potential disruption of these fundamental regulatory pathways in MND and suggests that the identified candidate MND genes are highly relevant to central nervous system (CNS) function and pathology.

## Discussion

This study represents an important exploration of the genetic architecture of MND, leveraging the extensive UK Biobank cohort to identify preliminary genetic risk factors. Our primary finding suggests that PTVs in 18 genes are significantly associated with an increased risk of MND, potentially expanding the known genetic landscape of the disease. The identification of *NEK1*, a gene previously established as an ALS-associated locus (Yao et al., 2021; Jiang et al., 2023), serves as a crucial internal validation, affirming the robustness and sensitivity of our analytical pipeline in detecting genuine disease-associated genes. Importantly, the remaining 17 genes represent new candidate associations that warrant extensive functional and replication studies.

To further strengthen the statistical evidence for these preliminary findings and exclude known major confounding factors, we performed stringent sensitivity analyses. These analyses, which excluded individuals with known pathogenic/likely pathogenic mutations for ALS/MND or *C9ORF72* GGGGCC repeat expansions, provided a highly refined set of genetic associations. Our results showed that 14 of the initial 18 genes remained significantly associated with MND risk, highlighting the statistical confidence in these specific findings. While four genes lost significance in sensitivity analysis, we remarkably identified six additional genes (*EPHX1, SELENOV, CDT1, ACTN3, USP16, and MAJIN*) whose PTVs were significantly enriched in MND cases. This extensive and rigorous validation process suggests a broad and heterogeneous genetic contribution to MND.

Our work on the UK Biobank is highly relevant to other population-based genetic studies. For instance, a recent study published in Brain (Gao et al., 2025) utilized the same UK Biobank cohort to estimate the high population risk of neurodegenerative disease in *C9ORF72* hexanucleotide repeat expansion (HRE) carriers, while also identifying the *UNC13A* genotype as a key genetic modifier. Our rigorous methodology, which includes the explicit exclusion of *C9ORF72* HRE individuals, aligns with the necessity of controlling for this major genetic factor, ensuring that the preliminary associations we report are independent of the most common cause of ALS/FTD and complement the findings on common variants and modifiers.

Our comprehensive functional enrichment analyses shed crucial light on the potential biological relevance of these newly identified genes. While KEGG pathway and GO-BP and GO-MF analyses did not yield significant enrichments, GO-CC analysis revealed significant enrichment in Collagen-Containing Extracellular Matrix and Cilium. The extracellular matrix (ECM) provides essential structural support (Theocharis et al., 2016), mediates cell–cell and cell-matrix interactions, and plays a crucial role in neuronal development, survival, and function (Fawcett et al., 2019). Alterations in ECM composition or integrity can impact neuronal migration, connectivity, and overall neuronal health, potentially contributing to neurodegeneration (Long and Huttner, 2019). Cilia, particularly primary cilia, are ubiquitous organelles found on most mammalian cells, including various types of neurons (Wang et al., 2024; Ma et al., 2022). They serve as crucial signaling hubs involved in diverse processes such as neuronal development, differentiation, and synaptic function (Tu et al., 2023). Growing evidence implicates ciliary dysfunction (ciliopathies) in a spectrum of neurological disorders (Khan et al., 2024). Our findings suggest that genetic variations impacting the integrity of the extracellular matrix or the proper function of cilia may predispose individuals to MND by compromising the supportive microenvironment or fundamental signaling pathways essential for motor neuron health and survival.

Furthermore, our investigation into the tissue-specific expression of the candidate genes provides compelling supporting evidence for their role in a CNS disorder. The analysis of DEGs showed a predominant enrichment of up-regulated DEGs in brain regions. Most notably, the total DEG analysis revealed that the brain hypothalamus exhibited the highest tissue specificity. This finding is particularly significant given the hypothalamus’s well-established roles in energy metabolism, autonomic function, and neuroinflammation, all of which are increasingly recognized as contributing factors to MND pathology (Chen et al., 2025; Miller and Spencer, 2014). This strong brain-specific expression pattern, particularly in a functionally crucial region like the hypothalamus, points towards a potential disruption of these fundamental regulatory pathways and provides biological plausibility for how genetic perturbations in these genes may directly contribute to the neurodegenerative processes observed in MND.

Our study benefits from the immense sample size and comprehensive WES data of the UK Biobank, providing unparalleled statistical power to detect rare genetic associations and ensuring robust control for confounding factors through advanced statistical methods like REGENIE (Mbatchou et al., 2021). The identification of a total of 20 candidate genes represents a substantial contribution to the field, suggesting an expansion of the genetic landscape of the disease. The rigorous sensitivity analyses, which excluded known genetic confounders, provide a high degree of confidence in the statistical nature of our findings. However, several limitations must be acknowledged. While the UK Biobank is vast, our findings are preliminary observations that mandate immediate and independent replication in diverse, large-scale MND cohorts to confirm these associations and assess their generalizability. Furthermore, while ALS is the most prevalent form, a critical limitation is our inability to precisely distinguish between MND subtypes (e.g., ALS, PMA, PLS) due to the nature of the algorithmically-derived outcomes (ICD codes) available in the UK Biobank. Therefore, we cannot comment definitively on the specific relationship between the identified gene PTVs and any single MND subtype. Moreover, the interpretation of rare variants regarding their precise effect size and penetrance remains challenging and requires further investigation. Crucially, the lack of detailed individual-level phenotypic data (motor and cognitive function) for our cases is a significant limitation, preventing us from exploring potential genotype–phenotype correlations or prognostic implications of the identified variants, which will be a key focus for future studies with clinical cohorts.

Building upon these compelling preliminary findings, future research should prioritize several key areas. Independent replication of these associations in diverse MND patient cohorts is paramount to establish their definitive role as genetic risk factors. Subsequently, in-depth functional studies are urgently needed to dissect the precise molecular mechanisms by which these genes contribute to motor neuron degeneration. This will involve using advanced cellular models (e.g., patient-derived iPSC motor neurons, CRISPR/Cas9-edited cells) and relevant animal models to assess their impact on critical cellular processes such as ECM integrity and ciliary function. Exploring the precise impact of specific PTVs within these genes on protein function and cellular phenotypes will also be critical. From a translational perspective, these novel genetic insights open new avenues for developing therapeutic strategies. The identified pathways suggest potential targets for pharmacological interventions that could modify the ECM or ciliary function. Ultimately, integrating these genetic findings with other multi-omics data (e.g., RNA-seq, proteomics, single-cell sequencing) will provide a more holistic understanding of their intricate roles in MND pathogenesis and pave the way for personalized medicine approaches.

## Data Availability

Publicly available datasets were analyzed in this study. This data can be found at: https://www.ukbiobank.ac.uk/.

## Footnotes

1
https://www.ukbiobank.ac.uk/learn-more-about-uk-biobank/about-us/ethics

2
https://www.ncbi.nlm.nih.gov/clinvar/
